# Survival Secrets: Unmasking the Factors Predicting Failure of Non-operative Management (NOM) in Splenic Injuries

**DOI:** 10.7759/cureus.47332

**Published:** 2023-10-19

**Authors:** Arun K Singh, Nemi Chandra J, Shivani B Paruthy, Vivek Belsariya, Sushila Choudhary

**Affiliations:** 1 General Surgery, Vardhman Mahavir Medical College, Safdarjung Hospital, New Delhi, IND

**Keywords:** splenectomy, trauma, blunt trauma abdomen, spleen, non-operative management

## Abstract

Background: Splenic injuries are common solid organ injuries resulting from blunt abdominal trauma in road traffic accidents. Very often, splenic injuries can be life-threatening. Earlier, splenic injuries were often dealt with surgical intervention, such as splenectomy. With the recognition of the immunological function of the spleen and possible complications of splenectomy surgery, such as overwhelming post-splenectomy infections (OPSI), there has been a recent trend for non-operative management (NOM).

Objective: To study the variables predicting failure of NOM in blunt abdominal trauma patients with splenic injury.

Methods: This is a retrospective study that includes 235 patients who presented to the Safdarjung Hospital emergency room (New Delhi, India) with blunt trauma abdomen and splenic injuries with or without associated injuries between January 2019 and December 2021. The data was entered in a Microsoft Excel spreadsheet (Microsoft Corp., Redmond, WA, USA). Categorical variables were expressed as frequencies and percentages. Pearson's chi-square test of association was used to determine if there is a relationship between two variables. A p-value of <0.05 was considered statistically significant.

Results: Out of 235 patients with blunt abdominal trauma and splenic injuries, 82 were hemodynamically unstable despite resuscitation and were taken up for emergency laparotomy. The remaining 153 patients, who were either hemodynamically stable or stabilized after adequate resuscitation, were managed on the lines of NOM. The number of patients with splenic injury in AAST grades 1, 2, 3, 4, and 5 was 36, 50, 40, 24, and three, respectively. Out of 153 patients, 130 (85%) were successfully managed by NOM, while eight (5%) had to discontinue NOM as they required surgical intervention. The failure of NOM (fNOM) is seen mostly with grade 5 injuries (2/2, 100%, p<0.01), followed by grade 4 (4/20, 20%) and grade 3 (2/37, 5.7%). The mean age in fNOM was 58.3 years, as compared to 42.2 years in the success of NOM (sNOM). All eight patients had multiple concomitant injuries, with femur fracture being the most common association in up to six patients (p<0.01), followed by liver injury in four patients. There were 15 mortalities, irrespective of AAST severity grade. All of these patients had associated concomitant injuries, with intracranial bleeding (n = 10, 32%, p<0.01) being the most common association, followed by femur fracture (n = 6, 20%) and liver injury (n = 5, 16%). Also, the cause of death was unrelated to splenic trauma (p = 0.67), with pulmonary embolism (n = 6, 40%, p<0.01) being the most common cause, followed by brain stem herniation (n = 5, 34%).

Conclusion: Non-operative management is a safe and efficient method for treating patients with splenic injuries who are hemodynamically stable or stabilized. The factors associated with fNOM include elderly age, a higher American Association for the Surgery of Trauma (AAST) grade of splenic injury, and associated concomitant injuries. Femur fracture was the most common concomitant injury present in cases where NOM failed, followed by liver injury. The presence of intracranial bleeds in these patients was a common association with mortality, irrespective of the grade of splenic injury.

## Introduction

A splenic injury from abdominal trauma is one of the most fatal solid organ injuries [1,2]. Non-operative management (NOM), which is employed by up to 70% of patients, has recently become the recommended mode of treatment [3]. With success rates above 80%, numerous studies have been conducted to support this treatment technique. [4]. Good patient selection is the most crucial requirement for the effectiveness of NOM. Hemodynamic instability despite sufficient resuscitation is the sole unequivocal contraindication for NOM in splenic injuries. [3]. The initial assessment of patients for hemodynamic instability, adequate resuscitation as per the advanced trauma life support (ATLS) protocol, and periodic reassessment should be the standard of care. The use of focused assessment with sonography in trauma (FAST) as a primary imaging modality will support the initial clinical assessment. The responders to initial resuscitation must be taken up for a detailed radiological study of the abdomen by contrast-enhanced computed tomography (CECT). Those patients who remain hemodynamically unstable despite adequate attempts at resuscitation are labeled as ‘non-responders’ and should be taken up for exploratory laparotomy.

Many decades ago, the majority of splenic trauma was managed by splenectomy. In recent times, with the introduction of newer resuscitation guidelines, advancements in imaging techniques, and angioembolization techniques, major surgical interventions like splenectomy and splenorrhaphy can be avoided. There has been an increasing trend towards spleen salvage procedures and the NOM of splenic injury patients, recognizing the immunological role of spleen in humans, erythrogenesis, and the possible complications after splenectomy, like overwhelming post-splenectomy infections (OPSI). Consequently, NOM has evolved into the preferred standard of care for hemodynamically stable or stabilized cases of splenic damage in institutions with adequate diagnostic facilities and intensive care units [3-5].

There are studies where 80% of traumatic spleen injury patients were successfully treated with NOM [5-7]. The failure rate of NOM can depend on multiple factors, including the grade of the splenic injury. Our goal is to research the elements that contribute to the failure of NOM (fNOM) in blunt splenic injuries and to evaluate the impact of NOM on splenic injuries. In recent years, the NOM of blunt splenic injury in adults has become increasingly popular. The factors that predict the effectiveness of NOM in patients with splenic damage are still subject to debate.

## Materials and methods

**Figure 1:**
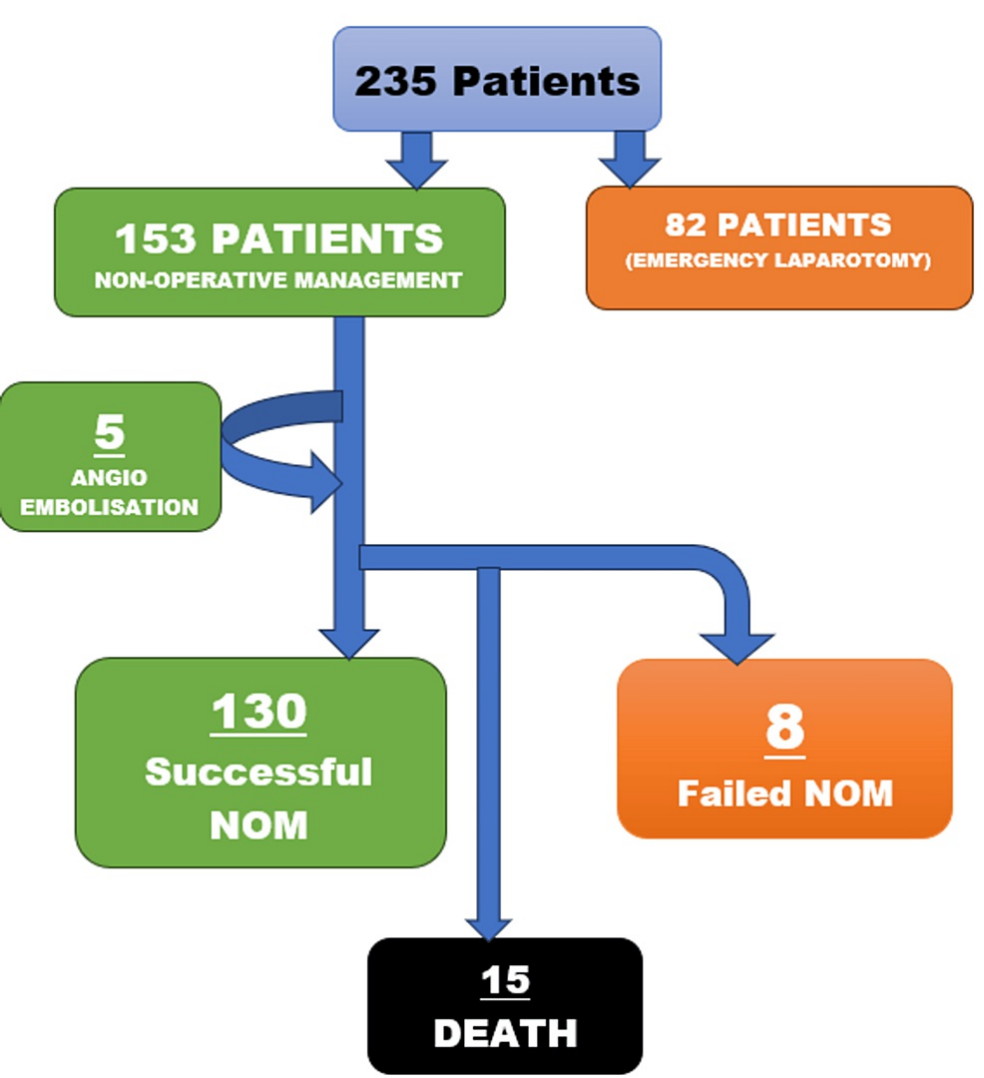
Methodology NOM: Non-operative management

A retrospective observational study was conducted to include over 235 patients who presented to the emergency room of Safdarjung Hospital, New Delhi, India, with blunt trauma abdomen and splenic injuries with or without associated injuries between January 2019 and December 2021 (Figure 1). Of these patients, 82 who were hemodynamically unstable despite resuscitation or had associated hollow viscus perforation were taken up for emergency laparotomy. The remaining 153 patients were either labeled as 'stable' or 'stabilized'. Stable patients were those who arrived at the emergency room in a hemodynamically stable state, i.e., systolic blood pressure greater than 100 mmHg and a heart rate less than 100 beats per minute. Stabilized patients included those who had to be resuscitated to achieve a hemodynamically stable state. Resuscitative measures included intravenous fluids, blood transfusion whenever necessary, injection of tranexamic acid, strict vital monitoring, etc. Later, these patients were managed per NOM. All patients were subjected to emergency FAST and were taken up for CT angiography of the abdomen. Patients were graded according to the American Association for the Surgery of Trauma (AAST) grading system for splenic injuries. The number of patients in grades 1, 2, 3, 4, and 5 was 36, 50, 40, 24, and three, respectively. Five patients were managed by angioembolization (three patients had an AAST grade 5 injury, one patient had grade 4, and another patient had grade 3).

All 153 patients under NOM were under strict vital monitoring, abdominal girth charting, six-hourly hemoglobin and hematocrit monitoring, strict bed rest, and input-output charting. Eventually, eight out of 153 patients developed hemodynamic instability and falling hemoglobin levels and were taken up for emergency laparotomies. The fNOM was noted in the early 48 hours of admission in four patients and another four patients between 48 and 96 hours.

This retrospective data collected over three years was entered into a Microsoft Excel spreadsheet (Microsoft Corp., Redmond, WA, USA). Statistical testing was conducted with SPSS Statistics version 28.0 (IBM Corp., Armonk, NY, USA). Continuous variables were presented as mean (SD) or median (IQR) for non-normally distributed data. Categorical variables were expressed as frequencies and percentages. Pearson’s chi-square test, or the chi-square test of association, was used to determine if there is a relationship between two variables. A p-value of <0.05 was considered statistically significant. A wide range of parameters were taken into consideration, such as the age of the patient, systolic blood pressure and heart rate at presentation, and concomitant injuries at the time of presentation in the emergency room. The fNOM of splenic trauma in eight patients was analyzed in detail to deduce the possible factors that led to it. The 15 mortalities were also studied in detail to analyze the possible association between concomitant injuries and the cause of death.

## Results

**Figure 2:**
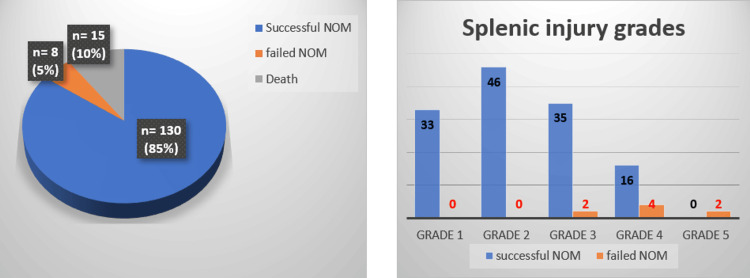
Outcomes NOM: Non-operative management

The number of patients with splenic injury in AAST grades 1, 2, 3, 4, and 5 was 36, 50, 40, 24, and three, respectively. Of the 153 patients, 130 (85%) were successfully managed by NOM. For 8 patients (5%), NOM had to be discontinued as they were intervened surgically. Grade 5 injuries (2/2, 100%) mostly resulted in fNOM, followed by grades 4 (4/20, 20%) and 3 (2/37, 5.7%). No fNOM) was reported in patients with splenic injuries of AAST grades 1 and 2 (Figure 2).

**Figure 3:**
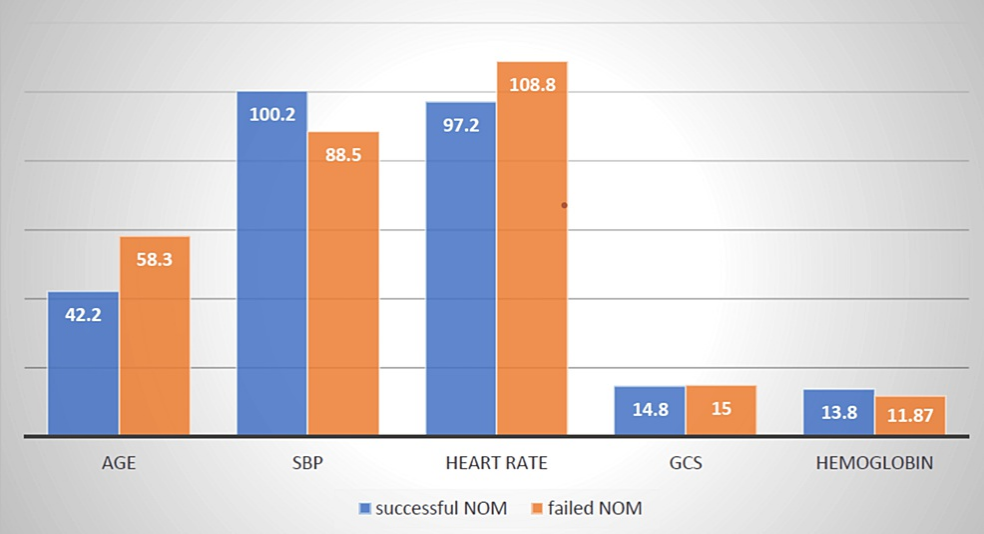
Variables on admission SBP: Systolic blood pressure, GCS: Glasgow coma scale, NOM: Non-operative management

On detailed analysis, six out of eight patients were over 50 years of age. The mean age in fNOM was 58.3 years, as compared to 42.2 years in successful NOM (sNOM). Retrospectively, seven out of eight patients under fNOM had to be stabilized by adequate resuscitation in the emergency room. The mean systolic blood pressure and heart rate at admission in fNOM were 88.5 mmHg and 108.8 beats per minute, respectively, as compared to 100.2 mmHg and 97.2 beats per minute, respectively, in sNOM (Figure 3).

**Figure 4:**
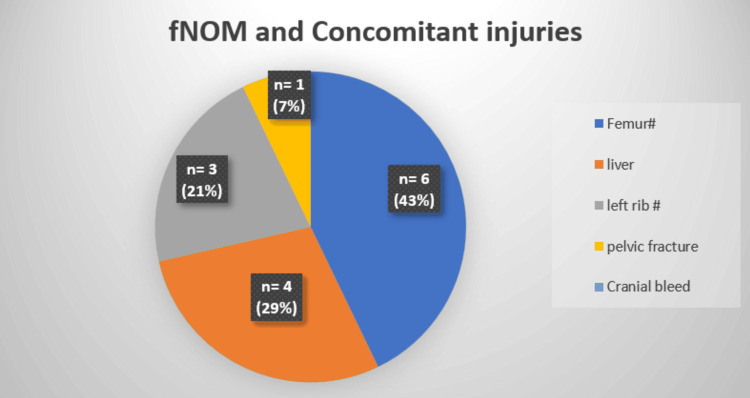
fNOM and concomitant injuries fNOM: Failure of non-operative management

All eight patients had multiple concomitant injuries, with femur fracture being the most common association in six patients (p<0.01), followed by liver injury in four patients (Figure 4, Table 1).

**Table 1:** Statistical analysis of variables in relation to fNOM A p-value <0.05 is considered significant. fNOM: Failure of non-operative management

Variables	Proportions	p-value	Inference
Elderly age		<0.01	Significant
Grade of splenic injury		<0.01	Significant
Concomitant injuries		<0.01	Significant
a. Femur fracture	43%	<0.01	Significant
b. Liver injury	29%	<0.01	Significant

**Figure 5:**
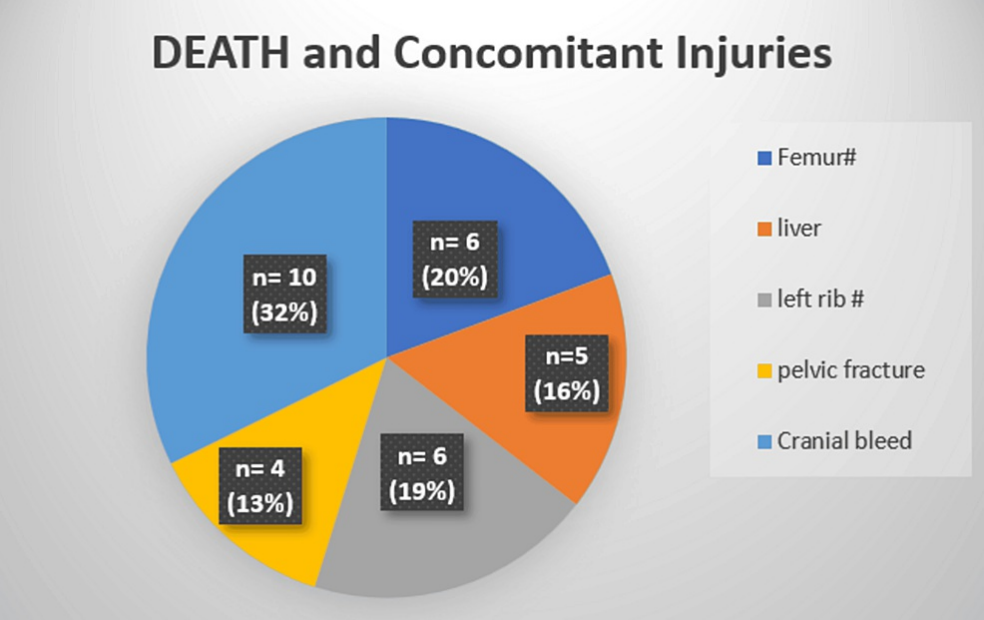
Death and concomitant injuries

**Figure 6:**
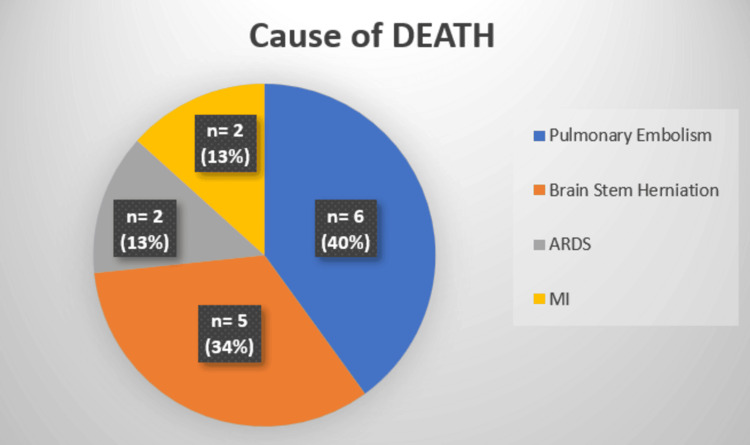
Cause of deaths ARDS: Acute respiratory distress syndrome, MI: Myocardial infarction

There were 15 mortalities (10%), irrespective of AAST severity grade. All of these patients had associated concomitant injuries (p<0.01); intracranial bleeding (n = 10, 32%, p<0.01) was the most common association, followed by femur fracture (n = 6, 20%) and liver injury (n = 5, 16%) (Figure 5). Therefore, the cause of death was unrelated to splenic trauma; pulmonary embolism (n = 6, 40%, p<0.01) was the most common cause, followed by brain stem herniation (n = 5, 34%), acute respiratory distress syndrome (ARDS) (n = 2, 13%), and myocardial infarction (n = 2, 13%) (Figure 6, Table 2).

**Table 2:** Statistical analysis of variables in relation to death A p-value of <0.05 is considered significant.

Variables	Proportions	p-value	Inference
Elderly age		<0.01	Significant
Grade of splenic injury		<0.67	NOT Significant
Concomitant injuries		<0.01	Significant
a. Intracranial bleed	32%	<0.01	Significant
b. Femur fracture	20%	<0.01	Significant
c. Liver injury	16%	<0.01	Significant
Cause of death		<0.01	Significant
a. Pulmonary embolism	40%	<0.01	Significant
b. Brain stem herniation	34%	<0.01	Significant

## Discussion

The spleen is the intra-abdominal organ that suffers blunt abdominal damage most frequently in cases of automobile crashes. Gruber et al. initially recognized the link between the spleen and immunological function in 1951 when they reported that an infant receiving therapy for idiopathic thrombocytopenic purpura encountered severe sepsis following splenectomy [8]. Zarrabbi and Rosner recorded 47 adult cases of serious infection following splenectomy for trauma. The results of their study displayed two significant findings: (1) the vast majority of serious infection cases occurred in the initial 10 years following splenectomy; however, some instances were recorded up to three decades ago; and (2) the majority of cases involved children, implying that kids this age could be particularly prone to sepsis after splenectomy [9]. As a consequence, spleen salvage treatments and the NOM of splenic damage in patients are becoming increasingly popular. Hemodynamic instability is the sole absolute contraindication [10].

Few studies point out that multisystemic trauma, significant brain damage, related injuries such as diaphragmatic injury and hollow viscus injury, age >55 years, and sick spleen are relative contraindications for NOM [11-14]. Low morbidity and mortality, avoidance of unnecessary laparotomies, minimal blood transfusions, shorter hospital stays, maintenance of immunological function, and prevention of OPSI are the primary benefits of NOM [15,16]. The predictive parameters for a sNOM, according to relevant studies [14,17,18], are as follows: hemodynamically stable or stabilized patients, need for blood transfusions of 4 units or less, age <55 years, early remission of splenic abnormalities, no loss of consciousness, and no brain trauma; absence of corresponding intra- or retroperitoneal injuries (upon abdominal CT scan) requiring surgical intervention; no guarding or rebound tenderness; and full restoration of bowel movements. One of the most crucial indicators of fNOM is the severity of the spleen injury. Per some study results, spleen injuries in grades 1 to 3 are regarded as low-grade, while patients with grades 4 through 5 are labeled high-grade. According to the updated scale, a grade 3 spleen injury is considered high-grade if it is additionally accompanied by another solid organ damage [8,19].

According to Peitzman et al. [20], NOM proved efficient in managing grade 1 to grade 5 spleen traumas in 75%, 70%, 49.3%, 16.9%, and 1.3% of patients, respectively. Retrospective research on the management and results of traumatic splenic damage over 15 years was done by Jesani et al. [21] in 2020 in a UK hospital. The study concluded that in patients with splenic trauma, selected non-operative therapy was effective. The prognosis of these patients can be improved by an integrated multidisciplinary diagnosis, standardized treatment, and therapeutic approach.

An investigation of 129 isolated splenic injuries from 2009 to 2016 was done retrospectively by Bagaria et al. in 2019. Patients were successfully treated non-operatively in 71.3% of cases. A splenectomy was later performed on three patients in the NOM arm, giving NOM an overall success rate of 96.8% [22]. Similar results have been observed in our study, concluding that the NOM of splenic injuries is an effective approach.

Elderly age, higher grades of splenic injury, and concomitant injuries are often associated with fNOM. The limitations of this study are its small sample size and the need to eliminate confounding factors before declaring the association of certain injuries with fNOM.

## Conclusions

Hemodynamically stable or stabilized patients with splenic injuries can be managed safely and effectively with NOM. Factors such as elderly age, a high AAST grade of splenic injury, and associated concomitant injuries are associated with fNOM. Femur fractures followed by liver injuries were the most common concomitant injuries present in cases where NOM failed. The presence of intracranial bleeding in these patients was a common association with mortality, irrespective of the grade of splenic injury.
